# Examining healthcare purchasing arrangements for strategic purchasing in Nigeria: a case study of the Imo state healthcare system

**DOI:** 10.1186/s12961-022-00844-z

**Published:** 2022-04-18

**Authors:** Charles Ezenduka, Eric Obikeze, Benjamin Uzochukwu, Obinna Onwujekwe

**Affiliations:** 1 Health Policy Research Group (HPRG), Department of Pharmacology & Therapeutics, College of Medicine, University of Nigeria Enugu Campus, Enugu, Nigeria; 2 Department of Health Administration & Management, Faculty of Health Sciences & Technology, College of Medicine, University of Nigeria Enugu Campus, Enugu, Nigeria; 3 Department of Community Medicine, College of Medicine, University of Nigeria Enugu Campus, Enugu, Nigeria

**Keywords:** Universal health coverage, Health financing, Purchasing functions, Strategic purchasing, Purchaser–provider split, Provider performance

## Abstract

**Background:**

Strategic healthcare purchasing (SHP), as a critical function of health financing, enhances the optimal attainment of health system goals through the efficient use of financial resources. Countries committed to universal health coverage (UHC) have made progress towards strategic purchasing through relevant reforms in their healthcare financing systems. This study examined the purchasing arrangements and practices in the Imo state healthcare system to track progress towards SHP committed to UHC.

**Methods:**

A critical review and analysis of healthcare financing schemes in Imo state, south-eastern Nigeria, was undertaken to assess their purchasing practices based on a descriptive qualitative case study approach. Relevant documents were collected and reviewed including in-depth interviews with stakeholders. Information was collected on external factors and governance, purchasing practices and other capacities of the state’s health financing schemes. The analytical framework was guided by comparing purchasing practices of the financing schemes with the ideal strategic purchasing actions (SPAs) developed by RESYST (Resilient and Responsive Health Systems), based on the three pairs of principal–agent relationships.

**Results:**

Healthcare purchasing in the state is dominated by the State Ministry of Health (SMOH) using a general tax-based and public health system, making government revenue a major source of funding and provision of healthcare services. However, purchasing of health services is passive and the stewardship role of government is significantly weak, characterized by substantial insufficient budgetary allocations, inadequate infrastructure and poor accountability. However, the health benefit package significantly reflects the needs of the population. As an integrated system, there is no purchaser–provider split. Provider selection, monitoring and payment processes do not promote quality and efficiency of service delivery. There is very limited institutional and technical capacity for SHP. However, the state recently established the Imo State Health Insurance Agency (IMSHIA), a social agency whose structure and organization support SHP functions, including benefit packages, provider selection processes, appropriate provider payment mechanisms and regulatory controls.

**Conclusion:**

Healthcare purchasing in Imo state remains mostly passive, with very limited strategic purchasing arrangements. The main challenges stem from the entrenched institutional mechanism of passive purchasing in the government’s health budgets that are derived from general tax revenue, lack of purchaser–provider split, and poor provider payment and performance monitoring mechanisms. The establishment of the social insurance agency represents an opportunity for boosting SHP in the state for enhanced progress towards UHC. Building capacity and awareness of the benefits of SHP among policy-makers and programme managers will improve the efficiency and equity of health purchasing in the state.

## Background

In a well-functioning healthcare system, the financing of healthcare is defined by three key functions: (1) revenue generation, which involves the mobilization and collection of funds from different sources; (2) resource pooling, where generated revenue is accumulated to ensure availability to the population in need; and (3) the purchasing function, which involves the transfer of pooled funds to healthcare providers for the provision of healthcare services [1–3].

Hence, as a core function of healthcare financing, purchasing provides a critical link between revenue generation and healthcare provision [3]. The purchasing function is used to achieve both efficiency and equity—through the allocation of resources for optimal health outcome (efficiency) and to the more critical need (equity). This makes purchasing an important financing function that links resources mobilized for universal health coverage (UHC) and effective delivery of quality healthcare services [3]. However, how the purchasing function is implemented has implications for achieving the desired objectives.

Healthcare purchasing can be implemented either passively or strategically [1]. In passive purchasing, funds are transferred to healthcare providers based on historical or predetermined budgets, without efficiency considerations—just paying bills when presented [2–4]. Strategic purchasing involves a continuous process of searching for the best ways to maximize health system performance, deciding which interventions should be purchased, how to purchase, and from whom to purchase [3, 5]. This entails applying the best methods to determine which healthcare services to buy (benefit package), from whom to buy (choosing the right service providers) and how to buy (provider payment mechanism)—in ways that maximize health system performance at the desired level [4]. This requires the evaluation of the health needs of the population, the planning and design of healthcare services, the qualification and selection of appropriate providers, and the incentivization and management of providers to ensure good performance [5].

Unlike passive purchasing, strategic health purchasing (SHP) is used to control costs and direct the purchasing of desirable quality services [4]. In this way, SHP interventions enhance the health system accountability and financial balance. In strategic purchasing, funds are transferred to healthcare providers in ways that incentivize them to seek efficiency, equity and quality of service delivery [2, 3]. This helps to achieve responsiveness, improved health outcomes and financial risk protection in healthcare delivery [2].

**Fig. 1:**
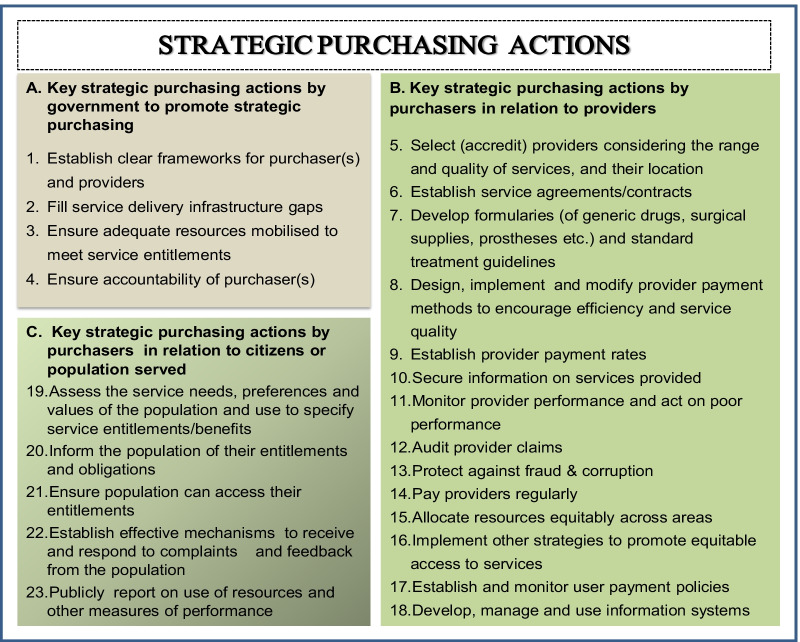
RESYST framework on ideal strategic purchasing actions [3]

The implementation of SHP is centred on three relationships which place the purchaser at the centre of the relationships, requiring the purchaser to engage actively with three main actors—the government (regulator), the healthcare providers and the citizens [3]. In this way, three arrangements are defined in the implementation of SHP: regulator–purchaser, purchaser–provider and purchaser–citizen. Within these arrangements are defined sets of strategic actions that ensure the optimization of resources to achieve desired outcomes. These actions are listed in Fig. 1 [2]. Consequently, the strategic arrangements imply the pursuit of three policy objectives associated with the actors in the relationships, namely government stewardship, improved provider performance and patient empowerment [5].

The ideal actions represent the responsibilities of the key actors under the arrangements for achieving the strategic objectives. As summarized in Table 1, the government’s stewardship roles are geared towards the provision of policy, institutional and regulatory frameworks to support strategic purchasing functions and practices (Fig. 1, actions 1–4). Purchasers’ roles with regard to providers and citizens include strategic selection/accreditation, incentivization and performance monitoring of healthcare providers to ensure quality and efficiency of service delivery; others include taking actions that represent the accountability and responsiveness of the purchaser to the comprehensive needs and preferences of the target population, thereby empowering citizens towards efficient service delivery [3].

**Table 1:** Summary of strategic purchasing actions

Government–purchaser axis
Governments should provide policy and regulatory frameworks that support strategic purchasing
Purchaser–provider axis
Purchasers should select and contract health service providers based on criteria including capacity and geographical distribution
Purchasers should require providers to ensure quality of service including through quality improvement mechanisms such as the use of standard treatment guidelines
Purchasers should incentivize provider performance through payment mechanisms and related incentives
Citizen–purchaser axis
Benefit packages should reflect the needs and preferences of the target population
Benefit packages should offer protection from financial catastrophe

*Source* Adapted from RESYST [3]

The implementation of a purchaser–provider arrangement is generally operated as either a contract or integrated system [2, 6]. Under the contract system, the organization purchaser enters into contract with service providers who are separate from the organization, such as when an insurance agency enters into contract with both public and private providers to provide healthcare services to its enrollees [2, 5]. However, when the purchaser agency uses its own healthcare providers, such as the health ministry, to purchase healthcare, it is said to operate an integrated system [2, 6].

Given its focus on efficiency, strategic purchasing has become the preferred option promoted to enhance the efficiency of resource utilization for optimal attainment of health system goals [1]. Consequently, in line with the current focus on strategic purchasing, health systems, especially in low- and middle-income countries (LMICs), have undertaken many reforms to strengthen their health financing systems towards strategic purchasing objectives [2, 7–9]. This has led to the establishment of a mandatory social health insurance model in many countries, including Nigeria, where most states have established or are in the process of introducing this mechanism which is designed to support strategic purchasing functions. Studies indicate that although these efforts are in place, purchasing practices remain mostly passive, with many factors limiting the implementation of strategic purchasing, such as unsatisfactory benefit packages, poor service quality and challenges with provider payment mechanisms [10–12]. Given the many challenges of the healthcare financing system in Nigeria, and efforts at improving efficiency such as through the promotion of the SHP functions, it has become necessary to track progress towards achieving this objective to inform policy for enhanced objectives.

The Imo State Ministry of Health (SMOH) is the principal provider of healthcare services in the state, playing the majority role as the state’s largest healthcare purchaser. The recently established Imo State Health Insurance Agency (IMSHIA) is expected to become a major player in healthcare delivery in the state, and hence the second major healthcare purchaser in the state. As the two main financing schemes in Imo state, little is known about how these purchasing arrangements and practices align with the SHP standards and the impact on the performance of the health system.

The paper provides new knowledge from a study that was conducted to examine the purchasing arrangements of the state’s health financing schemes. This was to help in understanding the extent to which purchasing arrangements and practices align with strategic purchasing functions to inform policy for the promotion of SHP for achieving UHC in both the state and similar contexts.

## Methods

### Study setting

Imo state in south-eastern Nigeria is one of the country’s most populous states, with an estimated population of 5 520 602 in 2017 [13], and a population density of 710 inhabitants/km^2^ by the 2006 census. The state is one of the Nigerian oil-producing states, with a gross domestic product (GDP) of $6.296 billion in 2017, and per capita GDP of $1140.58. The state economy is primarily dependent on agriculture and commerce, with a majority of the population (74%) engaged in agriculture.

The health sector operates a three-tier system comprising primary, secondary and tertiary levels of healthcare provision. The primary health level is now controlled by the Imo State Primary Health Care Development Agency (SPHCDA), in line with the Primary Health Care Under One Roof (PHCUOR) policy of the National Primary Health Care Development Agency (NPHCDA). The secondary level, which includes the general hospitals, is controlled by the State Hospital Management Board (SHMB). There are three tertiary healthcare facilities in the state: Imo State Specialist Hospital and Imo State University Teaching Hospital, run by the state government, and the Federal Medical Centre (FMC), which is controlled by the federal government [14].

The health sector suffers from significant underfunding, poor infrastructure and a dearth of human resources, overstretched by a rapidly expanding population [14–16]. There is high disease burden. Healthcare provision in the state is about equally shared between the public and private sectors, with a total of 1135 healthcare facilities [14]. However, most of the public secondary facilities are nonfunctional due to poor infrastructure and lack of human resources. High participation of the private sector in healthcare delivery has contributed significantly to the high levels of coverage of essential health services in the state. Routine health information is collected through the District Health Information System (DHIS2).

Health financing indicators in the state show four sources of healthcare funding, dominated by household out-of-pocket (OOP) expenditure at 92% of total health expenditure (THE), followed by the government at 7.6%, insurance at 0.2%, and donors providing 0.1%. THE stood at $578.93 million with $104.5 per capita in 2017 [15, 16]. To improve the financing and provision of healthcare, the state government established a social health insurance scheme (SHIS) in 2018 as part of a commitment towards achieving UHC. This was followed with the development of the Imo State Health Financing Policy & Strategy (HFP&S) in 2019, guided by the principles and values of UHC.

### IMSHIA

The IMSHIA was established by the Imo State Health Insurance Law 4 of 2018, to provide a legal backing for a SHIS for enhanced access to quality and affordable healthcare for all residents of the state [17]. IMSHIA is managed by a governing board, with a chairman appointed by the governor to oversee the affairs. An executive secretary is responsible for the day-to-day activities of the agency as head of the management team made up of six departments. A quality assurance department is responsible for accreditation and monitoring of service providers to ensure maintenance of service quality based on established guidelines. Sources of revenue include premiums and equity funds for indigent and vulnerable populations, including the federally allocated Basic Health Care Provision Fund (BHCPF). A benefit package of healthcare services is based on the explicit minimum service package, known as the Basic Minimum Package of Health Services (BMPHS), adapted from the national package according to the state’s needs and revenue, covering promotive, preventive, curative and rehabilitative care services. These are to be provided at primary and secondary healthcare facilities. A 10% copayment on medication is provided on generic-only policy. Accreditation/selection of service providers is based on a contractual arrangement for both public and private healthcare providers who sign agreements to provide a range and quality of services and their location. Providers are to be paid on a capitation and fee-for-service (FFS) basis, and on agreed rates, with strong regulatory and monitoring mechanisms to be undertaken on a quarterly basis to ensure delivery of services according to quality guidelines. Operational guidelines [18] provide a road map for the operation of the scheme. Key financing/service indicators are summarized in Table 1. Under the law, IMSHIA is also designated to undertake purchasing functions for the scheme. Currently in the early stages of operation, the mandatory insurance system is projected to become the main health financing scheme in the state to enhance progress towards UHC. Enrolment is ongoing, with about 1.8% of the population registered as of 2019 [16]. Many services are yet to be implemented and, hence, not available for assessment in this study prior to full operation.

#### Study design and framework

A qualitative case study approach was used to collect relevant data for conducting empirical analysis of the purchasing practices and activities of the two main financing schemes in the Imo state healthcare system. Examination included the organizational environment within which the agencies operate. Relevant documents were reviewed. In-depth interviews were conducted with key informants/stakeholders [2, 19]. Information on external factors and governance, purchasing practices, other capacities and resulting outcomes of the financing schemes were collected. Activities within this set of information were analysed for SHP functions. The analytical framework was guided by comparing the purchasing practices of the agencies with the standard SHP functions, based on the three pairs of principal–agent relationships—government–purchaser, purchaser–provider and citizen–purchaser [19]—including specific actions/activities within them.

## Conceptual framework

This is based on the framework developed in the study by Figueras et al. in 2005 [3] and the Resilient and Responsive Health Systems (RESYST) in 2014 [20], and applied in previous studies [2, 12, 19]. The framework outlines the main characteristics of the purchasing functions which describe three relationships between the purchaser and three key actors: the government, healthcare providers and the citizens [5]. Each of these relationships is defined by a set of ideal strategic purchasing actions (SPAs) which inform the performance of strategic purchasing functions to benefit the principals. This was therefore applied in this study to analyse the relationships between the two main purchasing schemes in Imo state, SMOH and IMSHIA, and the government, providers and citizens, respectively. The purchasing practices identified within each of these relationships were then compared with the ideal SPAs as described in the RESYST framework [3]. These actions were drawn from the literature and experiences on strategic purchasing including the understanding from the RESYST consortium members [2–5].

### Data collection

Data were collected between November 2019 and June 2020 from a review of relevant documents (Table 2) and the use of in-depth interviews with key informants from the SMOH and IMSHIA, and healthcare providers, who were purposively selected based on discussions with policy-makers [2].

**Table 2:** List of documents reviewed in the study

Number	Document
1	Imo State Health Account 2018
2	Imo State Health Financing Policy & Strategy 2019 (HFP&S)
3	State Auditor General reports 2014–2017
4	State Accountant General reports
5	State Public Performance Management Report 2019
6	Imo State Strategic Health Development Plan (SSHDP) II
7	Imo State Household Survey Report 2018
8	Imo Health Insurance Agency Operational Guidelines
9	Annual budget documents 2014–2017
10	Multiple Indicator Cluster Survey (MICS) 2016–2017
11	Nigeria Demographic & Health Survey 2018
12	Save One Million Lives (SOML) Implementation Guidelines
13	National Health Act (NHA) 2014
14	National Health Policy (NHP) 2016
15	Imo State Primary Health Care Development Agency (SPHCDA)
16	Imo State Public Expenditure Financial Accountability (PEFA) Report, World Bank March–June, 2011
17	Imo state Public Financial Management and Health Financing for Universal Coverage Report 2019
18	Imo State Health Insurance Agency (IMSHIA) Act 2018

Document search and reviews were conducted by two experienced researchers from the Health Policy Research Group (HPRG), University of Nigeria, Enugu Campus (UNEC). Reviewed documents included policy and legal documents, statutes, reports and operational guidelines. Websites (content) and grey literature were also accessed and reviewed. The reviewers extracted relevant information from the documents using the template provided by the Strategic Purchasing Africa Resource Centre (SPARC) team, which contains specific questions related to strategic purchasing functions and activities, in terms of government/external factors, purchasing functions, other capacities and outcomes, to be assessed for the extent to which they align with SHP objectives. Data from the reviewed documents were extracted into Excel standardized forms for assessment and summary of the collected information.

In-depth interviews were similarly conducted by the two researchers with 16 key informants comprising directors and senior and middle-level officers from both agencies (11 from SMOH and five from IMSHIA, and made up of seven women and nine men, Table 3). The respondents were selected based on their knowledge of healthcare financing and purchasing arrangements within their agencies, to obtain relevant information on healthcare purchasing practices. Information was obtained using the same data extraction template that contains specific questions on purchasing functions and practices. Prior to the interviews, prospective respondents were contacted by phone and email and were sent information on the purpose of the study, including their involvement and right to participate. Each participant signed a written informed consent form before the interview. The interview was guided by topics which were informed by the objectives of the study and conceptual framework. Researchers also sought clarification from respondents regarding information obtained from reviewed documents. The audio-recorded interviews, with field notes by researchers, lasted between 40 and 60 minutes each. Further meetings were held with respondents as feedback for their views on study findings. All data collected were presented and extensively discussed as a team of researchers to collectively agree on information/findings to address possible bias for enhanced validity. Overall data were further shared with the SPARC review team for their comments to inform final analysis.

**Table 3:** Respondent summary

Respondent	SMOH	IMSHIA	Total
	Number interviewed		
Executive directors	1	1	2
Senior managers	3	2	5
Middle-level managers	5	2	7
Healthcare providers	2	–	2
Others	–	–	–
*Total*	*11*	*5*	*16*

*SMOH* State Ministry of Health, *IMSHIA* Imo State Health Insurance Agency

### Documents reviewed

#### Data analysis

Analysis of data was guided by the conceptual framework described by RESYST and Figueras et al. [3, 20], based on the characteristics and relationships between the purchasing agency and the three actors in healthcare purchasing, namely the government, regulators and the citizens [2, 3]. Hence, data collected on the characteristics and activities of the SMOH and IMSHIA and their respective relationships with the Imo state government, healthcare providers and citizens were analysed to determine the extent to which these characteristics and actions aligned with SHP functions. Analysis included identification of gaps and opportunities for enhanced implementation of SHP in the state.

### Ethical considerations

Ethical approval was obtained from the Health Research Ethics Committee of the University of Nigeria Teaching Hospital, Enugu, Nigeria. Each participant signed a written consent form before the interview. The consent form contained brief information on the purpose of the study, participants’ rights and measures to be undertaken to ensure confidentiality of information given.

### Results

Findings are presented in this section to reflect the outcome of the analysis of the purchasing arrangements between the respective agencies (SMOH and IMSHIA) and each of the three actors, and the extent to which purchasing practices/actions represent strategic purchasing.

Table 4 highlights the key characteristics of the two purchasing schemes in Imo state which provide enablers for implementing strategic purchasing functions.

**Table 4:** Summary characteristics of the financing schemes

Indicator/purchasing function	Imo SMOH	IMSHIA
Model	Supplier side	Partner–agent model
Sources of revenue	Government budget 100%: tax revenue and other sources	Prepayment scheme (premium payment), BHCPF, equity funds, government subventions
Target population	Total population	Imo state citizens/residents who pay the appropriate premium and meet criteria for enrolment
Total expenditure per beneficiary per year	₦31 966 (US$ 104.5) 2017	Not available yet
Requirement for entitlement	Registration card as citizen, OOP	(1) Payment of monthly ₦1000 premium (2) Employees of public and organized private sector (3) Employees of informal sector (4) Indigents and vulnerable persons
Coverage	Not established	1.8%, representing proportion of Imo state population covered to date (2019)
Premium rate	Not determined	₦12 000 per annum full benefit
Benefit packages	Minimum packages of preventive, curative and promotive care services	Minimum package of preventive, curative and promotive care services
Purchasing agency	SMOH (SHMB, SPHCDA)	IMSHIA/No third-party administrator
Health service providers	Public providers	Public and private providers
Provider payment mechanism	Salary, line-item budget	Capitation (primary care), FFS (secondary and tertiary care)
Budget execution	State and local government levels	The agency

*SMOH* State Ministry of Health, *IMSHIA* Imo State Health Insurance Agency, *BHCPF* Basic Health Care Provision Fund, *OOP* out-of-pocket, *SHMB* State Hospital Management Board, *SPHCDA* State Primary Health Care Development Agency, *FFS* fee-for-service

### Government: SMOH arrangement

#### Policy and legal framework

A number of policy and legal frameworks exist in the state in the form of health policy and legislation by the government that guide the purchasing and provision of healthcare services by the SMOH. This represents an ideal action and responsibility of the government in the relationship with the SMOH as a purchasing agent (SPA 1, Fig. 1). This includes the decisions on which services to provide, who should provide service and how payments should be made, as stipulated in the policy and legal documents of the state adapted from the guidelines of the National Health Policy and National Health Act [14, 21]. For instance, the National Health Policy and National Health Act from which the states derive directions include the implementation of the BMPHS that includes promotive, preventive, curative and rehabilitative healthcare services [14, 22]. These services are to be provided across the different levels of healthcare delivery (primary, secondary and tertiary health facilities) according to the jurisdictions of local, state and federal governments.

*The ministry adopted the Basic Minimum Package of Healthcare Services in line with national guidelines adapted to the Imo state situation to reflect the state’s disease burden and population preferences. These services are supposed to be provided at all the public health facilities in the state.* (Senior-level manager, SMOH)

As part of the commitment towards UHC, the government recently introduced a policy framework in the form of the HFP&S [16], developed to guide adequate generation of healthcare revenue, effective pooling and strategic purchasing for healthcare delivery over a 5-year period, supporting SHP functions and practices. This represents another ideal action that supports SHP, in addition to the IMSHIA Law of 2018.

Payment of providers is undertaken through salary and line-item budget/resource allocation for service delivery, subject to the Public Procurement Act of 2007 and Public Finance Management Act regulations, which stipulate allocation of budgets for service delivery [23]. However, within the SMOH, donor-supported equity programmes such as the Save One Million Lives (SOML) are implemented with incentive measures that are linked to achievement of established targets. Regular monitoring measures are implemented to ensure achievement of targets, making the process strategic. This has contributed to increased access to maternal and child health (MCH) services in Imo state [14, 16, 24].

*SOML is a donor-supported project from the federal government designed to improve MCH care in the state. The project is implemented through the SMOH, using the public healthcare providers to achieve targets of MCH services. Providers are given special training with other incentives that drive achievement of specified targets.* (Senior-level manager, SMOH)

#### Poor service delivery infrastructure

As a key government stewardship responsibility (SPA 2), health delivery infrastructure in Imo state is poor, reflected in acute lack of human resources in all cadres, inadequate public health facilities and lack of essential health commodities, leading to huge infrastructure gaps. This is attributed in large part to poor budgetary allocations and release, as well as poor prioritization in the budget process [16, 25]. This huge health infrastructure gap limits effective healthcare delivery in the state, including the implementation of donor-supported programmes for vulnerable groups which are implemented through the SMOH.

*We don’t have enough facilities in the state to cater for huge needs of the population. Available facilities are in very poor condition, lacking in essential equipments and commodities. Acute lack of health workers such as doctors and nurses has remained a recurring problem, affecting effective provision of healthcare services in public health facilities.* (Middle-level manager, SMOH)

#### Inadequate budget allocation and poor disbursement

Mobilization of adequate resources for healthcare is a key SPA (SPA 3) of the government. However, healthcare in Imo state is grossly underfunded. Reviewed documents showed very poor government budget allocations for healthcare to meet the healthcare needs of the state. Analyses show that the government budget for healthcare averaged below 4% of the total budget over the 8-year period of 2010–2017, well below the Abuja target of 15% [15, 16]. Similarly, government health expenditure as a proportion of THE was 1.8% in 2017, while as a percentage of GDP the state government spent 2.8% on healthcare, below the 5% threshold recommended for LMICs [15]. Other challenges in the budget allocations include the continuous budget cuts and reallocation to other departments, further constraining the limited resources available for service delivery [16, 25, 26]. Finally, delays and unpredictability in the release and disbursement of resources from the government treasury to the ministry is another challenge that undermines the availability of resources for service delivery [25, 26].

*Budgeting processes in Imo state [are] quite poor. Approved budget for health is always inadequate to meet the needs of the state, and release of the budget is always delayed without knowing when the money will be available for service delivery. What is released is well below the approved figure, with incessant cuts and reallocations.* (Senior-level manager, SMOH)

Implementation of donor-supported equity programmes targeting vulnerable groups increases the availability of funding for improved coverage and access to priority services for target groups such as maternal and child health services.

#### Limited financial autonomy

The responsibility of the government to provide for adequate resource mobilization (SPA 3) for improved healthcare purchasing includes providing for the use of internally generated revenue (IGR) by providers as a form of financial autonomy to enhance purchasing decisions for improved service delivery [3]. This autonomy is very limited in the Imo SMOH, where the Procurement Act allows for only limited use of IGR/user fees at the facilities—40% at the secondary health facilities for maintenance—while the rest is returned to the ministry [23, 25]. This remains very inadequate, leading to poor maintenance of infrastructure and poor-quality services. Primary healthcare facilities are not allowed the use of IGR for maintenance, while allocated funds remain inadequate. Meanwhile, the budgeting process does not adequately consult with health facilities, hindering effective planning and budgeting. This consequently undermines purchasing through poor prioritization of needs for the health department and restricted access to financial resources [5].

#### Poor or weak accountability of the SMOH

The responsibility of the government includes ensuring that the SMOH as a purchaser is accountable for their performance and financially accountable (SPA 4) [3]. While the study showed requirements for this reporting, there was little or no information on the implementation of this process that demonstrated performance and financial accountability of the SMOH to the government. Performance accountability requires the SMOH to ensure that healthcare provision and activities in the state conform to established guidelines which derive from the state’s annual development plans as outlined in the State Strategic Health Development Plan (SSHDP), in terms of the goals, implementation plans and performance indicators [14, 16]. This includes ensuring that progress is made towards achieving national health performance indicators on service delivery. The study showed that the SMOH hardly undertakes or adheres to these requirements, and there was no reported consequence for nonadherence. For financial accountability, the SMOH should regularly account for the use of public funds to the government (through the Budget Office and Auditor General) and the general public. The provision for this process is stipulated in the Public Finance Management Act which provides the basis for budgeting [25], with consequences for noncompliance. However, reports indicated significant noncompliance of this accountability requirement, with no reported sanctions. Evidence shows a lack of relevant technical and financial capacity in the state to perform this function. Donor-supported programmes in the SMOH use established guidelines which are effectively monitored and evaluated to ensure quality and performance accountability [27].

### SMOH relationship with healthcare providers

#### There is poor implementation of quality assurance mechanisms

Although it is part of the responsibilities and actions of the purchaser to accredit providers based on the range and quality of services to be provided to inform service contracts, this was not the case with the Imo SMOH and the providers due to lack of a purchaser–provider split. Consequently, SPAs 5 and 6 were not met (Fig. 1). As an integrated system where the SMOH used their own public health providers, there were no contracts or responsibility for equipping and funding the facilities (public providers), and thus they were unable to sanction poor performance. On the establishment of treatment guidelines and formularies (SPA 7), these are provided for common illnesses and essential drug lists to standardize service delivery and guide commodity management across all levels of healthcare [5, 21, 22]. Healthcare providers are required to use the treatment guidelines and essential drug lists to ensure quality of service delivery. However, these are not always available in all the facilities, and where available, they are often not adhered to [14, 16]. Moreover, there was no evidence of the existence of therapeutic committees and quality improvement teams, to monitor the implementation of these tools.

With regard to monitoring of provider performance and possible sanction of poor performance to ensure continuous delivery of quality services (SPA 11), findings show that provision is made for quarterly monitoring supportive supervision but this is rarely implemented by the ministry. Providers are designed to be assessed by monitoring teams, for quality of service provision using health indicators and adherence to standard treatment guidelines [14]. Findings show poor or lack of regular performance of this function by the SMOH. This appeared to be due mainly to lack of commitment and funding for such activities [14]. The system lacks clear framework and reporting structures for monitoring and evaluating provider performance.

*Monitoring units from the SMOH are expected to undertake quarterly visits to assess performance using health indicators and adherence to guidelines. This is rarely implemented. They don’t consider this important.* (Middle-level manager, SMOH)

With the donor-supported programmes in the ministry, providers are selected and given special training for effective delivery of selected services. Measures of quality assurance include the use of implementation guidelines and quantitative data reporting. Regular monitoring of provider performance is undertaken using supportive supervision and sanction for noncompliance to ensure adherence to performance guidelines and quality of service delivery [25].

#### Provider payment mechanism is inadequate and irregular

For SPA 8, in which purchasers are expected to establish a payment mechanism that would encourage efficiency of provider performance, the SMOH operates the line-item budget and monthly salary payment systems to public health providers for health service provision. They are similarly expected to set payment rates, adequate for enhanced performance (SPA 9). The line-item budgets which are based on historical patterns of expenditure are often considered insufficient by the providers for meeting the needs of service delivery. As a result, providers ration services and charge user fees for services designed to be free, in order to sustain the services [2]. Salaries are paid according to fixed rates determined by staff categories and scales, not linked to performance. This often leads to complaints of inadequacy and is worsened by constant delays in payment and promotion and limited chances for increasing salaries [25, 26]. Strategic purchasing under this arrangement requires that providers are paid regularly (SPA 14). The poor salary rates and frequent delays lead to demotivation and frequent strikes or industrial actions by the health workers in the state, impacting negatively on the quality of services.

*As I am talking to you now, we have not been paid salary for up to 3 months since this year. This has remained a regular occurrence in this state, worse than other states in the region. To make it worse, we are often paid less than our actual salaries. It has been very frustrating working in this state.* (Middle-level manager SMOH)

Within the donor-supported programmes such as the Save One Million Lives (SOML) in the SMOH, incentives are introduced that motivate healthcare providers to deliver on established targets [27], representing a strategic measure to improve provider quality and efficiency of service delivery. Regular monitoring measures are implemented to ensure achievement of objectives. This has also contributed to increased access to MCH services in Imo state [14, 16, 24].

#### Health information management system is weak

The purchasing agency needs a strong, stable and effective information management system for successful performance of their purchasing functions (SPAs 10 and 18). This requires well-developed information and communication technology to facilitate the sharing and use of information to ensure the delivery of quality and effective healthcare [2, 3]. Purchasers need this process in collecting relevant information on health provider activities to enable evidence-based planning and decision-making. However, this system in Imo state is weak, as the state is still grappling with the DHIS2 system. Health-related information is paper-based, with the use of registers. Access to electronic information systems is lacking, as providers do not have computers [14, 16]. The implication or result is poor reporting and generation of information needed for performance monitoring as well as for support and decision-making. Consequently, there is generally poor data quality in the health system due to poor reporting, inability of the SMOH to undertake regular supervision and data checks, and shortages of recording staff. A challenge was noted previously in multiplicity of reporting due to requirements of vertical programmes such as HIV/AIDS and malaria programmes, which undermined the quality of reporting [14, 16]. This is, however, being addressed at the time of this study with the adoption of the integrated DHIS2 system to harmonize information processing in the healthcare system.

*Even when donor agencies/partners provide these electronic supports, they are hardly utilized due to lack of relevant infrastructure such as internet/network services among others.* (Middle-level manager, SMOH)

#### Inequitable allocation of resources across service areas

Allocation of resources across service and geographical areas according to priority needs of the population (SPA 15) is important for achieving the equity objective of strategic purchasing. This requires the purchasing agency to allocate a greater share of resources to preventive or primary healthcare services than to curative healthcare, as well as equitable distribution of services and health workers across geographical areas to ensure effective coverage [3]. Findings show that curative healthcare received a disproportionately larger share of funding than preventive care over the prior 8 years, 2010–2017 [15, 16]. Expenditures on primary and other priority healthcare services were significantly lower during the period. A greater proportion of the healthcare budget in the ministry was devoted to recurrent expenditure [16]. As previously described, the healthcare system is characterized by a dearth of health workers, resulting in many underserved areas lacking in staff and relevant health services [14]. In addition, the study did not identify any other strategies in place for the promotion of equity in the SMOH (SPA 16). As an important element of equity, measures of financial risk protection should be in place to protect the population from financial catastrophe [3]. The study did not document any discernible measure for implementing or monitoring user payment policy (SPA 17).

#### Poor financial audit system

One of the responsibilities of a purchasing agency is the regular auditing of provider claims to protect against fraud and corruption (SPAs 12 and 13) [3]. This is also provided for in the state’s Public Finance Management Act, which requires special audit units within and outside the ministry to ensure transparency and accountability [23, 25, 26]. However, in Imo state, evidence shows a very dysfunctional public finance management system, reporting gross misconduct and general lack of due process [25]. Meanwhile, as an integrated system, there is no submission of claims, but financial statements are required to be submitted in line with the budget policy. Audits are rarely conducted, despite being required on a quarterly basis. There are reported high levels of corruption. Meanwhile, there were no reported cases of sanction against defaulters who did not adhere to the financial requirements [25].

### SMOH–citizen arrangement

#### Poor needs assessment of citizens

As a key responsibility associated with the purchasing required for the development and revision of health services benefit packages to be delivered to citizens, a purchaser is required to conduct assessments of the needs of the population (SPA 19), to ensure that their preferences and values are appropriately captured in the services to be purchased and delivered. These must be conveyed to citizens to inform them of their entitlements and obligations [3] (SPA 20). There was no evidence of this process in the state by either the respondents or reviewed documents, in terms of public engagement of the ministry or its agents with the public on health service delivery relevant to their needs. However, the benefits package adopted by the state is in line with the national health policy which developed the BMPHS, which is based on the common healthcare needs of the Nigerian population [21, 22].

#### Service entitlements were poorly delivered

While purchasers are expected to ensure that citizens have access to health service entitlements (SPA 21) in terms of the availability of preventative, promotive, curative and rehabilitative care in all public primary and secondary health facilities, as recommended in the statute documents [22], these services are not comprehensively available in all the facilities [15]. Due to poor infrastructure, lack of adequate manpower and very limited financial resources, none of the public health facilities offers a comprehensive range of required services in the state. This limits citizens’ access to care, resulting in OOP payments for services, especially in secondary care, where user fees can be higher, further limiting access for citizens already burdened by poverty and financial constraints.

#### Lack of feedback and complaint mechanisms

The strategic purchasing function of the purchasing agency includes the establishment of public mechanisms (SPA 22) for providing feedback and complaints from citizens regarding services received from providers and the system. This includes the use of direct communication lines with facility managers, civil society organizations (CSOs), assembly members and others, as well as suggestion boxes and social media platforms [3]. The study documented no evidence or report of any of these actions from the SMOH as the purchasing agency, either from the respondents or from the general public. Structures for this process do not exist. The citizens are not even aware that such mechanisms or structure should exist. This limits the capacity for change and improvement in service delivery, undermining the accountability of purchasers to the citizens.

#### The SMOH does not demonstrate accountability to the citizens of Imo state

Public reporting of the use of resources and other performance measures by the purchaser is another responsibility of the agency to citizens (SPA 23) in order to demonstrate openness in public financing. This is performed through public participation and clear fiscal reporting. There was no report of public participation in any budget-making process and no dissemination or access to relevant financial documents or reports of implementation to the public [25].

### IMSHIA purchasing practices and arrangements

#### Government: IMSHIA relationship

Purchasing arrangements between IMSHIA and other actors in the Imo state healthcare system largely conform to strategic purchasing functions. Document reviews and interviews revealed the existence of appropriate legal and policy frameworks for implementing purchasing functions by the IMSHIA [17, 18]. These include decisions regarding which services to provide, who should provide service and how payments should be made, as stipulated in the IMSHIA law [17]. Benefit packages which are explicit comprise promotive, preventive and curative services, covering communicable and noncommunicable diseases. They are provided as the BMPHS in line with national policy at all healthcare facilities [22]. Cost-sharing is provided only for prescribed medicines at health facilities, where 10% copayment is made by enrollees, and does not apply to other services [17, 18].

*IMSHIA is an agency established by law, supervised by government through the Imo SMOH, but directly responsible to the governing board, which is appointed by the governor. It has three key responsibilities: resource mobilization, risk pooling and strategic purchasing.* (Senior-level manager, IMSHIA)

Revenue sources and generation mechanisms of the insurance agency make for adequacy, predictability and pooling of resources for healthcare provision. The funding mechanism also provides adequate autonomy for providers for enhanced purchasing decisions for improved service delivery. Implementation outcomes of these functions will be determined after full operation of the agency, which is currently in the early stages. Achievement of the required strategic objectives of these arrangements will be influenced by the government commitments towards filling the wide infrastructural gaps already existing in the state, as well as fulfilling other responsibilities.

### IMSHIA relationship with healthcare providers

Actions that inform IMSHIA’s relationship with healthcare providers are consistent with strategic purchasing functions. While measures for these actions are in place, many are yet to be implemented given the early stages of operation. As a contract system, IMSHIA, through the relevant department, selects and accredits both public and private healthcare providers based on the range and quality of services to be delivered, to inform service contracts [18] (SPA 5 and 6). Among other basic requirements, providers are expected to provide 24-hour service, ensure no discrimination among enrollees, stock generic medicines, maintain basic medical ethics, treat patients with dignity and open accounts with commercial banks. In addition, private providers are required to register with the SMOH and must be located within the state before being accredited. There are clinical and practice guidelines as well as an essential drug list to inform provision of quality and efficient services (SPA 7). Regular monitoring tools and measures are in place to ensure adherence to performance quality (SPA 11). The agency undertakes quarterly visits to providers for quality assessment [18].

*IMSHIA is involved in accreditation of service providers, periodic monitoring of quality of services provided, incentivization of providers and other quality assurance-related oversight.* (Senior-level manager, IMSHIA)

The agency operates a mixed payment system, where primary providers are paid prospective monthly capitation, FFS is used to pay secondary and tertiary care providers, and per diem is used for hospital bed space (SPAs 8 and 9). These are linked to performance, with monitoring mechanisms in place to influence quality performance by providers.

Health management information in the agency is undertaken through the DHIS2, which is both electronic and paper-based (SPAs 10 and 18). Providers are required to submit relevant information on their activities to the agency for planning and decision-making [18]. However, the process is currently mostly paper-based due to limited access to electronic information systems, as healthcare providers lack computers in addition to poor internet service in the state.

The agency maintains a financial audit arrangement to ensure transparency and accountability (SPA 12). Healthcare providers are required to submit their reimbursement claims on a monthly basis (within 30 days), which are vetted to protect against fraud and corruption (SPA 13). Payment is made within 40 days (SPA 14). Sanctions are in place for defaulters, as payments not submitted after 90 days will not be entertained. Implementation outcome will become known after full operation of the agency has been achieved.

### IMSHIA relationship with citizens

IMSHIA adopted the national policy guidelines on the provision of the BMPHS at health facilities accredited by the agency. This was based on the disease burden in Nigeria, adapted for Imo state, to take into account the prevailing local disease burden and morbidity pattern in Imo state, as well as on the acceptability, effectiveness/cost-effectiveness and equity of delivery [14, 18]. Findings show that the agency, currently in the introductory stages of operation, is yet to establish mechanisms for demonstrating accountability and responsibility to the public along the four elements of this relationship with citizens (SPAs 19–22). Evidence was lacking regarding communication with the public on availability and access to service entitlements, availability of complaint and feedback mechanisms for providing complaints and feedback on citizens’ experiences with service delivery and possible suggestions, and demonstration of financial accountability of the IMSHIA to the citizens (SPA 23). However, reports indicated that during registration, enrollees are provided with public telephone numbers to enable them share information on their experiences and concerns regarding the service delivery issues, in the form of complaints and feedback.

*Enrollees are given public phone numbers on enrolment to enable them seek for clarification on the service delivery as well as concerns they may have on their participation and experiences. In the near future we intend to implement mechanisms for providing other means of communication with the public.* (Middle-level manager, IMSHIA)

## Discussion

The paper presents new knowledge on the purchasing practices in the Imo state healthcare system, to show the extent to which purchasing arrangements represent strategic purchasing functions, using the SPAs that define purchasing arrangements between the purchasing agencies and the three actors in the purchasing relationships. The purchasing practices of both the Imo SMOH and the state insurance agency, IMSHIA, were analysed. The SMOH, as the largest healthcare provider in the state, is the dominant purchaser of healthcare for the citizens. The SMOH operates the integrated purchasing system using only their public providers for health service provision.

The findings showed that there was very little strategic purchasing activity along the three purchasing relationships. Examination of the government–SMOH relationship which establishes the stewardship and regulatory roles of government in healthcare purchasing showed that the performance of these roles was quite weak. Strategic purchasing requires that government develop policies that support SHP functions to ensure that providers achieve the desired objectives [2, 18]. While there are policy and legal frameworks that guide purchasing of healthcare services by the SMOH using policy and legal instruments, these are rarely enforced. Furthermore, as the only source of financing for healthcare, government budget allocation for healthcare is very limited and unpredictable, constituting a major barrier to achieving strategic health objectives. The negative impact of underfunding for healthcare is far-reaching, cutting across SHP functions and activities, leading to poor service delivery infrastructure and the consequences of poor quality and inefficiency of service delivery.

As a public/tax-based revenue system, the SMOH lacks the purchaser–provider split by operating the public integrated system where purchasers and providers are not organizationally separate [2]. This undermines the strategic purchasing principles, as the SMOH does not perform its role as a purchaser by ensuring quality through the selection of providers that meet defined standards of quality. This is an important part of strategic purchasing function that enables providers to deliver quality and efficient services based on their ability [2, 28].

Given the absence of a purchaser–provider split in the SMOH, the provision of a clear system for monitoring provider performance to ensure achievement of quality improvement would be necessary [3, 5] if strategic objectives are to be achieved. This would also require the presence of a robust health information system with timely sharing of information between the ministry and providers, in addition to proper financial and risk management systems [3, 28]. However, while the SMOH has some measures in place for monitoring provider performance, there is poor implementation and lack of a robust information system, which in essence undermines strategic purchasing objectives. Similarly, the integrated public system of the SMOH lacks a purchaser–provider split, limiting the financial autonomy of providers and consequently the ability of the providers to respond to service delivery needs. Similar findings were reported in Kenya [2]. In strategic purchasing arrangements, providers need financial autonomy to be able to respond to service needs and requirements for efficiency, improved performance and accountability, by limiting direct government influence on purchasing decisions [29].

As in most public systems, provider payment in the SMOH is implemented through salary and line-item budgets which are not linked to performance, indicating passive purchasing. This means that providers are not incentivized for quality improvement and efficiency of service delivery. This was similarly reported in 2017 in a public integrated purchasing system in a nearby state in Nigeria [11], as well as in studies in Kenya and South Africa [2, 30]. Salary payment is known to undermine health provider productivity, motivation and quality of care [31], while line-item budgets limit flexibility of resource allocation by both purchasers and providers [2, 5, 32]. Unlike the SMOH, the use of mixed payment methods at IMSHIA aligns with strategic purchasing principles designed to influence providers to pursue quality and efficiency of service delivery.

The results show that the IMSHIA operates the public and private contract system with established stewardship and regulatory roles of government in healthcare purchasing, in line with strategic purchasing objectives as provided for in the state’s health insurance agency law [17]. The sources of funding, which cut across premium payment, employer/employee contribution, equity funds, BHCPF and so on, guarantee sufficient and predictable funding for sustainable healthcare provision. Providers are contracted based on range and quality of healthcare services. The results of these strategic features following effective implementation would be expected to achieve strategic purchasing objectives.

However, IMSHIA operates contract arrangements with both public and private providers based on a range of quality and services to be provided. Agreements are signed to achieve the desired performance objectives. Monitoring mechanisms are in place to positively influence providers to pursue quality, efficiency and accountability [18]. Sanctions are also in place for poor performance. Agency operation is still early for result assessment, and effective implementation will ensure achievement of performance objectives for improved health outcomes.

The study identified no purchaser–citizen arrangement required for public accountability and responsibility of the SMOH as a purchaser to the population. There was no reported means of public participation between the ministry and the people. The citizens or beneficiaries do not have clear channels for providing timely feedback to the purchasers at either the SMOH or IMSHIA. The four elements of this relationship are lacking in the SMOH—no means of assessing citizens’ needs and preferences, lack of delivery of entitlements, no public complaint and feedback mechanisms, and no financial accountability—and these altogether undermine the strategic purchasing objectives. While these actions were also not implemented in the newly established IMSHIA, there are reported plans to establish such a mechanism in the near future. In its early stage of operation, IMSHIA has provided telephone numbers to enrollees to encourage the sharing of relevant information with the agency.

The findings of this study underscore the need to undertake significant reforms in the Imo state healthcare system to enhance effective implementation of strategic purchasing functions. Therefore, drawing from the findings, this study makes the following recommendations leveraging on opportunities identified in the state to boost SHP in Imo state. Achieving desired objectives depends primarily on the state government’s expressed commitment towards SHP for UHC. This commitment needs to be practically demonstrated through the strengthening of government stewardship and regulatory roles, undertaking adequate reforms in the system to establish structures that support SHP, increasing funding for healthcare, improving infrastructure and addressing manpower needs, and ensuring and enforcing regular performance monitoring for accountability and efficiency of service delivery. The impact of these measures on the overall success of strategic purchasing for UHC cannot be overemphasized.

Adequate and sustainable funding remains a critical requirement for achieving the required standards for SHP objectives. The state government needs to increase funding for healthcare in Imo state, which has remained very low and unpredictable over the years, limiting effective provision of healthcare in the state [16]. Expansion of fiscal space for healthcare financing is urgently needed to increase revenue mobilization for healthcare.

Given the poor state of health infrastructure, which is a major barrier to effective healthcare delivery, the government should strengthen health infrastructure by making appropriate investments for the development and revitalization of healthcare facilities to enable effective service delivery that addresses the comprehensive needs of the population. This should also include the possibility of collaboration with the private sector to leverage shared resources, especially for highly specialized skills and expensive equipment [2].

The health system should be reformed to introduce elements of purchaser–provider split between the SMOH and healthcare providers, to enhance the capacity of the purchaser to enforce provider accountability. The selection of healthcare providers should be explicit, based on stipulated criteria that ensure the delivery of quality service according to performance standards, for which they become accountable to the purchasers. This should be backed by enforcement of measures that enhance provider performance monitoring to ensure providers’ adherence to quality and efficiency guidelines.

The provider payment mechanism needs to be reviewed to introduce elements that motivate providers to seek efficiency and accountability. The use of fixed salary payment could be supported with a FFS specialist programme, which is currently used in the delivery of donor-supported programmes in the SMOH, with proven results. The line-item budgets could be reformed to target performance-based mechanism such as capitation for primary healthcare [2]. This would provide required financial autonomy to providers for improved resource allocation and service delivery, a key objective in strategic purchasing.

A robust health information system is central to achieving strategic purchasing objectives. Given the state of this system in the state, which lacks robust features, the health management information system needs to be strengthened to enhance evidence-based planning and decision-making. The electronic component of the information system should be strengthened through adequate provision of computers and internet services to health facilities to facilitate information-sharing between the SMOH and healthcare providers. Provision of dashboards, harmonization of information systems and similar measures will boost information management. Healthcare providers should be incentivized for timely submission of quality information. The use of financial incentives for compliance and sanctions for noncompliance can be employed to achieve this objective [2]. Provider payment mechanisms demand better and accurate data, which in turn stimulates improvement in the information system [33].

The lack of public participation and complaint and feedback mechanisms in the SMOH undermines strategic purchasing objectives for public accountability and responsibility. Government should recognize this and put measures in place as part of the requirements for monitoring and evaluating the performance of purchasers and providers, such as providing direct communication lines with facility managers, CSOs and assembly members, including suggestion boxes and social media.

It is recommended that government and its partners introduce capacity development programmes for implementing SHP. The system lacks adequate capacity for implementing strategic purchasing at both the SMOH and IMSHIA. Government needs to provide opportunities for regular training workshops for relevant stakeholders at both the ministry and the insurance agency, to enhance implementation of SHP.

The study noted the opportunities that exist within and outside the country for collaboration with experts for effective implementation of SHP in the state. Collaboration is needed with local and international partners and experts on strategic purchasing to share relevant information and expertise for enhanced implementation of SHP.

The findings show that the development of the state’s first HFP&S was part of the government’s demonstration of commitment towards achieving UHC, designed to ensure adequate fund mobilization for effective delivery of quality, efficient and equitable healthcare services through strategic purchasing, and hence an important policy tool that supports SHP functions and objectives. The effective implementation of the policy recommendations will boost strategic purchasing in the state.

These key strategic measures contribute to making the health system more resilient to respond to disease outbreaks, especially given the current COVID-19 pandemic. A robust electronic information system with surveillance features provides online real-time tracking services for prompt identification and management of diseases for better control, for which other strategic measures described above would be already in place as recommended.

### Strengths and limitations

For logistical reasons, our study did not collect data from citizens as key actors in the strategic arrangements. However, relevant information from the key informants and reviewed documents was adequate to inform our findings, in terms of lack of relevant communication channels with citizens; hence this will not affect the findings. Future studies would consider collecting citizens’ perspectives regarding these actions and related outcomes when fully implemented by the schemes. Similarly, the study did not collect some activity data in the framework due to non-implementation, especially from IMSHIA. These actions will be evaluated in a future study for comprehensive assessment of SHP in the state after full implementation.

One strength of this study lies in the use of a qualitative approach (a case study) to achieve analytical generalizability (other than statistical), by exploring a system to provide insights into strategic purchasing practices in Nigeria/Imo state in particular, with broader lessons for policy-makers in similar settings, on how their financing systems can become more strategic.

## Conclusion

Healthcare purchasing in Imo state remains mostly passive, with limited strategic purchasing functions. The SMOH is the dominant purchaser for healthcare in the state, followed by the social insurance agency IMSHIA, currently in the early stages of operation. Other agencies with purchasing functions within the ministry include the SPHCDA and HMB. The stewardship role of government is weak, characterized by an inadequate framework for SHP, insufficient and unpredictable healthcare budget, inadequate infrastructure and poor purchaser accountability, which constitute impediments to achieving SHP objectives. The absence of a purchaser–provider split and the poor provider performance monitoring and payment mechanisms result in failure to promote quality and efficiency of service delivery. Consequently, service delivery is suboptimal. There is limited institutional and technical capacity for SHP given the structure and expertise for SHP in the state. Adequate policy, legal and regulatory frameworks for implementing SHP need to be put in place and enforced. The establishment of the social health insurance scheme, IMSHIA, with a projected increase in citizen enrolment, represents major progress towards SHP. Expansion and strengthening of the insurance agency and effective implementation of the HFP&S, among other recommendations, present opportunities for boosting strategic purchasing for healthcare in the state. Further evaluation will be needed to determine whether SHP is appropriately implemented through IMSHIA and whether this translates to improved healthcare outcomes.

## Abbreviations

CSOs: Civil society organizations
HFP&S: Health Financing Policy & Strategy
HPRG: Health Policy Research Group
IMSHIA: Imo State Health Insurance Agency
LMICs: Low- and middle-income countries
UNEC: University of Nigeria, Enugu Campus
RESYST: Resilient and Responsive Health Systems
SHP: Strategic healthcare purchasing
SMOH: State Ministry of Health
SPARC: Strategic Purchasing Africa Resource Centre
SPHCDA: State Primary Health Care Development Agency
UHC: Universal health coverage
